# Hybrid Coronary Revascularization as a Safe, Feasible, and Viable Alternative to Conventional Coronary Artery Bypass Grafting: What Is the Current Evidence?

**DOI:** 10.1155/2013/142616

**Published:** 2013-04-03

**Authors:** Arjan J. F. P. Verhaegh, Ryan E. Accord, Leen van Garsse, Jos G. Maessen

**Affiliations:** Department of Cardiothoracic Surgery, Maastricht University Medical Center, P. Debyelaan 25, P.O. Box 5800, 6202 AZ Maastricht, The Netherlands

## Abstract

The “hybrid” approach to multivessel coronary artery disease combines surgical left internal thoracic artery (LITA) to left anterior descending coronary artery (LAD) bypass grafting and percutaneous coronary intervention of the remaining lesions. Ideally, the LITA to LAD bypass graft is performed in a minimally invasive fashion. This review aims to clarify the place of hybrid coronary revascularization (HCR) in the current therapeutic armamentarium against multivessel coronary artery disease. Eighteen studies including 970 patients were included for analysis. The postoperative LITA patency varied between 93.0% and 100.0%. The mean overall survival rate in hybrid treated patients was 98.1%. Hybrid treated patients showed statistically significant shorter hospital length of stay (LOS), intensive care unit (ICU) LOS, and intubation time, less packed red blood cell (PRBC) transfusion requirements, and lower in-hospital major adverse cardiac and cerebrovascular event (MACCE) rates compared with patients treated by on-pump and off-pump coronary artery bypass grafting (CABG). This resulted in a significant reduction in costs for hybrid treated patients in the postoperative period. In studies completed to date, HCR appears to be a promising and cost-effective alternative for CABG in the treatment of multivessel coronary artery disease in a selected patient population.

## 1. Introduction

Coronary artery bypass grafting (CABG) is considered to be the “gold standard” in patients with multivessel disease and remains the treatment of choice for patients with severe coronary artery disease, including three-vessel or left main coronary artery disease [1]. The use of CABG, as compared with both percutaneous coronary intervention (PCI) and medical therapy, is superior with regard to long-term symptom relief, major adverse cardiac or cerebrovascular events and survival benefit [1–4]. However, because of the use of cardiopulmonary bypass and median sternotomy, CABG is associated with significant surgical trauma leading to a long rehabilitation period and delayed postoperative improvement of quality of life [5]. An alternative “hybrid” approach to multivessel coronary artery disease combines surgical left internal thoracic artery (LITA) to left anterior descending coronary artery (LAD) bypass grafting and percutaneous coronary intervention of the remaining lesions [3, 6–8]. Ideally, the LITA to LAD bypass graft is performed in a minimally invasive fashion through minimally invasive direct coronary artery bypass grafting (MIDCAB) [9]. This hybrid approach takes advantage of the survival benefit of the LITA to LAD bypass, while minimizing invasiveness and lowering morbidity by avoiding median sternotomy, rib retraction, aortic manipulation, and cardiopulmonary bypass [3, 8, 10–14]. The purpose of the hybrid approach is to achieve complete coronary revascularization with outcomes equivalent to conventional coronary artery bypass grafting, while ensuring faster patient recovery, shorter hospital stays, and earlier return to work due to lower morbidity and mortality rates. 

 Angelini and colleagues reported the first hybrid coronary revascularization (HCR) procedure in 1996, and several patient series using hybrid coronary revascularization have been published since then [3]. These series support the above-mentioned presumptions and indicate that the hybrid approach is a feasible option for the treatment of selected patients with multivessel coronary artery disease involving the left main. Moreover, the introduction of drug-eluting stents (DESs) with lower rates of restenosis and better clinical outcomes may make hybrid coronary revascularization a more sustainable and feasible option than previously reported [9, 15]. 

 Nevertheless, this hybrid approach has not been widely adopted because practical and logistical concerns have been expressed. These concerns implicate the need for close cooperation between surgeon and interventional cardiologist, logistical issues regarding sequencing and timing of the procedures, and the use of aggressive anticoagulant therapy for percutaneous coronary intervention that may worsen bleeding in the surgical patient [7, 14, 16].

This review aims to clarify the place of hybrid coronary revascularization in the current therapeutic armamentarium against multivessel coronary artery disease. First, the patient selection for the HCR procedure is clarified. Second, the results of previous patient series using the hybrid approach are summarized and interpreted. Finally, the cost effectiveness of the HCR procedure is analysed.

## 2. Materials and Methods

### 2.1. Search Strategy

The MEDLINE/PubMed database was searched in January 2012 using the medical subject headings (MESH) for “coronary artery disease” and “angioplasty, balloon, coronary” combined with the following free-text keywords: “multivessel coronary artery disease,” “minimally invasive coronary artery bypass,” “percutaneous coronary intervention,” and “hybrid coronary revascularization”. One hundred seventy-seven articles matching these search criteria were found, and the search for additional papers was continued by analysing the reference lists of relevant articles.

### 2.2. Selection Criteria

**Figure 1:**
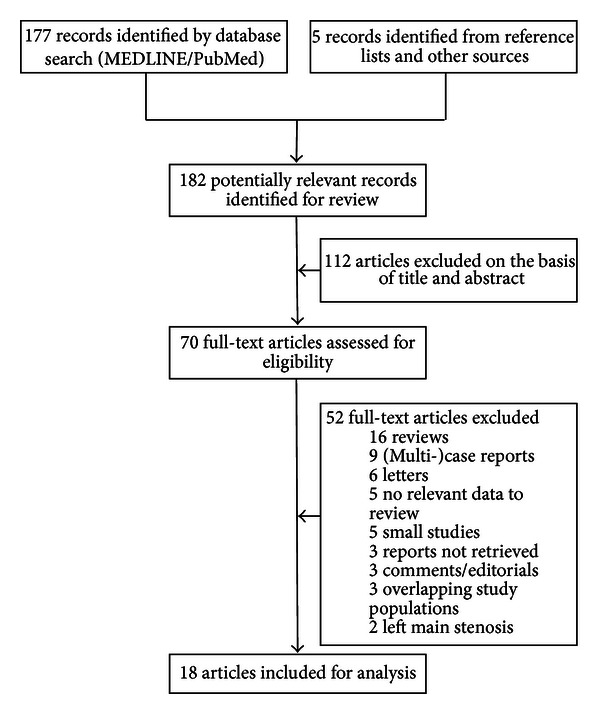
Study selection.

Randomized controlled trials, nonrandomized prospective and retrospective (comparative) studies were selected for inclusion. Publications in languages other than English were excluded beforehand. Letters, editorials, (multi)case reports, reviews, and small studies (*n* < 15) were also excluded. Studies examining the HCR procedure for multivessel coronary disease were included, while studies investigating the HCR procedure for left main coronary stenosis were excluded. Authors and medical centres with two or more published studies were carefully evaluated and were represented by their most recent publication to avoid multiple reporting of the same patients. A total of eighteen included studies remained eligible for analysis after applying these in- and exclusion criteria (Figure 1).

### 2.3. Review Strategy

The primary outcome measures were in-hospital major adverse cardiac and cerebrovascular events (MACCEs), packed red blood cells (PRBCs) transfusion rate, LITA patency, hospital length of stay (LOS), 30-day mortality, survival, and target vessel revascularization (TVR). Secondary outcome measures were intensive care unit (ICU) LOS and intubation time, as only a limited number of studies reported these outcome measures. In addition, the period of time between PCI and LITA to LAD bypass grafting and the cost effectiveness of HCR were examined. The long-term LITA patency was not included as an outcome measure, since only a limited number of studies report this outcome measure in a clear and concise manner.

In-hospital major adverse cardiac and cerebrovascular events were defined as postoperative stroke, myocardial infarction (MI), or death during hospital stay. Only the Fitzgibbon patency class A (widely patent) was considered as a patent LITA to LAD bypass graft, while the Fitzgibbon patency class B (flow limiting) and C (occluded) were defined as a nonpatent LITA to LAD bypass graft. Hospital LOS was defined as the number of days spent in hospital from operation to discharge. If the need for repeated revascularization involved a coronary artery initially treated with either bypass grafting or PCI, this repeated revascularization was considered to be target vessel revascularization. 

 One observer extracted all available outcome measures of each article and a second observer checked and supervised the first observer thoroughly. When an article did not disclose one or more of these outcome measures or reported medians and ranges as central tendency instead of means and standard deviations, the study was excluded from the analysis of that particular variable.

### 2.4. Statistical Analysis

The results were analysed using IBM SPSS Statistics 19 software (IBM Inc., Armonk, NY, USA). Continuous data were presented as mean and standard deviation (SD), while categorical data were expressed as numbers and percentages. 

## 3. Outline and Interpretation of the Results of HCR

Nine hundred seventy patients undergoing HCR procedures were included for analysis (Tables 1 and 2) [6, 7, 11–14, 17–28]. The most important findings are reported below. 

### 3.1. Patient Selection

The classical indication for an HCR procedure is multivessel coronary artery disease involving LAD lesion judged suitable for minimally invasive LITA to LAD bypass grafting but unsuitable for PCI (type C), and (a) non-LAD lesion(s) (most of the time right coronary artery (RCA) and/or circumflex coronary artery (Cx) lesions) amenable to PCI (type A or B) [7, 11, 12, 14, 17, 18, 20, 22, 23, 26–28]. High-risk patients especially with severe concomitant diseases (e.g., diabetes mellitus, malignancies, significant carotid disease, severely impaired LV function, and neurological diseases), who are more prone to develop complications after cardiopulmonary bypass and sternotomy, might benefit from the circumvention of CPB and sternotomy [11, 18, 20, 22–24].

Exclusion criteria for HCR consist of contraindications to minimally invasive LITA to LAD bypass grafting or PCI. LITA to LAD bypass grafting in a minimally invasive fashion requires single-lung ventilation and chest cavity insufflation. Therefore, HCR procedures are contraindicated in patients with a compromised pulmonary function (i.e., forced expiratory volume in one second less than 50% of predicted) and a small intrathoracic cavity space [14, 27, 28]. Moreover, patients with a nongraftable or a buried intramyocardial LAD, history of left subclavian artery and/or LITA stenosis, morbid obesity (BMI > 40 kg/m^2^), and previous left chest surgery are not well suited for minimally invasive LITA tot LAD bypass grafting [14, 20, 22, 27, 28]. Conditions rendering PCI unsuitable include peripheral vascular disease precluding vascular access, coronary vessel diameter smaller than 1.5 mm, tortuous calcified coronary vessels, fresh thrombotic lesions, chronic totally occluded coronary arteries, extensive coronary involvement, chronic renal insufficiency (serum creatinine ≥ 200 *μ*mol/L), and allergy to radiographic contrast [7, 14, 18, 20, 22, 27, 28]. Finally, haemodynamic instability, need for a concomitant operation (e.g., valve repair or replacement), and decompensated congestive heart failure are regarded as exclusion criteria [7, 17, 20, 22, 27, 28]. 

### 3.2. Timing of the HCR Procedure

The best timing of the interventions remains a matter of debate. Three HCR strategies can be distinguished: (I) performing PCI first, followed by LITA to LAD bypass grafting or (II) vice versa; (III) combining LITA to LAD bypass grafting and PCI in the same setting in a hybrid operative suite. In the included studies, staged HCR procedures (I and II) were applied much more frequently than simultaneous procedures (III). 

In a “staged” procedure, in which PCI and LITA to LAD bypass grafting are carried out at separate locations and/or different days, both interventions can be performed under ideal circumstances (in a modern catheterization laboratory and modern operating room, resp.) [11, 18, 29]. However, patients have to undergo 2 procedures, while they remain incompletely revascularized and at risk for cardiovascular events for an extended period of time [14, 29].

When PCI is performed first, a staged procedure takes place with an unprotected anterior wall, which could pose serious health risks in case the LAD lesion is considered the culprit lesion [13]. In addition, LITA to LAD bypass grafting is performed after aggressive platelet inhibition for prevention of acute (stent) thrombosis, which might lead to unnecessary postponement of following operation or may cause a higher than expected rate of bleeding [12, 13, 21, 29]. Moreover, stent thrombosis is risked after reversal of surgical anticoagulation and is related to the inflammatory reaction after cardiac surgery [13]. Furthermore, the opportunity for quality control of the LITA to LAD bypass graft and anastomosis by a coronary angiogram is lost and, therefore, this strategy requires a reangiography [12, 13]. These repeat control angiograms increase overall healthcare costs unnecessarily and decrease cost effectiveness [12]. 

Nevertheless, the potential advantages of this strategy are threefold. First, revascularization of non-LAD vessels provides an optimized overall coronary flow reserve, thereby minimizing the potential risk of ischemia and myocardial infarction during the LAD occlusion for LITA to LAD bypass grafting [6, 12]. Second, it is possible for the interventional cardiologist to fall back on conventional CABG in case of a suboptimal PCI result or major PCI complications. However, failure of PCI leading to emergency conventional CABG has become extremely rare with decreasing incidence since the introduction of coronary artery stenting [12, 20, 29–32]. Furthermore, this strategy allows HCR in patients with the immediate need for PCI in a non-LAD target and no immediate possibility for emergency bypass surgery [11, 24]. Critical stenosis in the right coronary artery (RCA) or the left circumflex coronary artery (LCx) or difficult PCI targets are considered as clear indications for a “PCI first” approach because these patients can undergo conventional CABG in case of PCI failure [11].

When the LITA to LAD bypass graft is performed first, antiplatelet therapy is routinely started after surgery to prevent antiplatelet-related bleeding complications during surgery and is present at time of PCI [6, 13, 27]. These antiplatelet agents can be administered long term, which is mandatory for preventing stent thrombosis. Moreover, the quality control of the LITA to LAD bypass graft and anastomosis can be performed simultaneously without a further angiogram [6, 12, 13, 18, 20, 23, 25, 26, 29]. In addition, PCI is performed in a “protective” environment with a revascularized anteroseptal wall, which probably reduces the procedural risks and gives the interventional cardiologist the ability to approach lesions that would be quite challenging without a revascularized LAD [13, 20, 25, 26, 29]. However, patients undergoing this strategy could require a second, much higher-risk, surgical intervention due to complications of the PCI [13, 23, 25]. Finally, the cardiac surgeon has to be aware of possible intraoperative ischemia during this HCR strategy because the collateral, non-LAD vessels are unprotected. 

Nevertheless, combining the two procedures in one stage under general anaesthesia in a specific hybrid-operating room, which combines the potential of catheterization and cardiac surgery, has advantages compared with staged HCR procedures [7, 14, 25, 28]. This simultaneous approach represents a single procedure that achieves complete revascularization, while minimizing patient discomfort and reducing the need for anaesthetics [12, 14, 18, 20, 28]. This approach eliminates logistic concerns about timing and sequence of two separate procedures and maximizes patient satisfaction [7, 14, 25, 28]. Moreover, the quality of the LITA to LAD bypass graft and anastomosis can be confirmed immediately by an intraoperative angiogram, which enables direct revision of the LITA to LAD bypass graft [18, 25]. Complications and difficulties during PCI or MIDCAB can be dealt with immediately in the same setting by conversion to conventional, open-chest CABG [25]. 

This procedure also has its own drawbacks. Perioperative haemorrhage can become a problem because full antiplatelet therapy and incomplete heparin reversal are necessary instantly after MIDCAB to prevent a transient “rebound” increase in thrombin formation associated with stent thrombosis and ensure an optimal intraoperative DES placement [7, 14, 18]. Besides, off-pump surgery may give rise to hypercoagulability and increased platelet activation during the early postoperative period, which is associated with an increased risk of stent thrombosis [33]. This makes antiplatelet management an important safety issue in HCR. Therefore, a modified antiplatelet protocol and careful patient selection seem appropriate, especially in one-stop HCR, in order to minimize the risk of stent thrombosis without increasing perioperative bleeding risk. A tried and tested protocol of dual antiplatelet therapy (DAPT) includes continuous use of aspirin (100 mg/day) until the operation day and intraoperative administration of a loading dose clopidogrel (300 mg) via a nasogastric tube after confirming LITA graft patency, followed by a maintenance dose of 75 mg/day for 12 months [34]. However, caution is required when using DAPT, since reversal agents for clopidogrel and aspirin are not available. Moreover, newer more potent antiplatelet agents, like prasugrel and ticagrelor, should be reserved exclusively for selected cases (high risk of stent thrombosis) and managed with even more care, since the clinical experience with these newer antiplatelet agents is limited in cardiac surgery and the bleeding risk may be increased. Furthermore, intraoperative collaboration and communication among cardiac surgeons, interventional cardiologists, and anaesthesiologists should be outstanding and ongoing to optimize continuity of care [11, 14]. Currently, this simultaneous procedure is used in only a few centres, and some authors state that this might be caused by the need to possess catheterization laboratories outfitted to accommodate cardiac surgery or hybrid operating rooms equipped with a mobile coronary angiography C-arm or permanent fluoroscopic equipment [7, 13]. 

The latter is reflected in the small number of patients undergoing a simultaneous procedure in our sample of included studies [7, 13, 14, 18, 24, 25, 28]. Expansion of other percutaneous and hybrid procedures like “hybrid AF ablation” may help to make these hybrid, multipurpose operating rooms more common in the future. However, staged HCR procedures could offer a more realistic alternative for many institutions without a so-called hybrid operating room, and this is supported by the fact that staged HCR procedures are applied much more frequently than simultaneous procedures in the included studies [6, 11–13, 17–24, 26, 27]. 

Tables 3 and 4 present the period of time between both procedures in a staged HCR strategy, and this period of time varied notably from 0 to 180 days. Therefore, some patients remained incompletely revascularized and were in theory at risk for cardiovascular events for a considerable length of time, while complete myocardial revascularization should be the main goal of treatment in patients with multivessel coronary artery disease. Moreover, Delhaye et al. found that PCI with clopidogrel preloading can be performed within 48 hours of LITA to LAD bypass grafting without increasing the bleeding risk [26]. In addition, Zenati et al. performed PCI zero to four days after LITA to LAD bypass grafting without increasing the PRBC transfusion requirements, while lowering the hospital length of stay (2.7 ± 1.0 days) [17]. The mean hospital length of stay was 5.5 ± 1.8 days (range: from 2.7 to 8.2 days), and hospital length of stay seems not to be influenced by the HCR strategy used (Table 2).

### 3.3. Surgical Techniques in Relation to Outcome Measures

As shown in Table 1, the surgical techniques for LITA to LAD bypass grafting have evolved continuously since the introduction of the HCR procedure in 1996 by Angelini et al. Most of the initial patient series performed the LITA to LAD bypass graft in a minimally invasive fashion carrying out a mini-thoracotomy on the anterolateral chest wall in imitation of Angelini et al. [3, 7, 12, 17–19]. In this so-called minimally invasive direct coronary artery bypass (MIDCAB) approach, the LITA is harvested under direct vision using specially designed LITA retractors. The anastomosis to the LAD is performed with 8-0 or 4-0 Prolene sutures on the beating heart (without CPB) with the help of mechanical stabilizers. In more recent patient series, the LITA was identified and harvested thoracoscopically or robotically, which decreased rib retraction, chest wall deformity, and trauma [11, 14, 21, 22, 27]. This approach significantly minimizes the typical thoracotomy-type incisional pain and wound complications of conventional MIDCAB, while optimizing graft length and retaining the reliability of manually sewn LITA to LAD anastomosis [21, 22]. Some teams prefer to place the LITA bypass graft to the LAD through a ministernotomy (inversed L-shaped or reversed J-shaped), which makes it possible to switch to full sternotomy in case complications may occur during the original operation [20, 23, 28]. Nevertheless, this surgical technique increases surgical trauma and, therefore, may raise morbidity and mortality. In addition, some centres even decided to perform the LITA to LAD bypass graft through a full sternotomy on the beating heart (off-pump CABG), thereby further increasing invasiveness [6, 25, 26]. If the LITA bypass graft is placed on the LAD through a sternotomy on the arrested heart (on-pump CABG), circumvention of CPB is lost too [6, 25, 26]. Thus, both on-pump and off-pump CABG can be seen as suboptimal procedures to carry out the LITA to LAD bypass graft. This might explain the higher MACCE rates found by Zhao et al. and Delhaye et al. and the high 30-day mortality discovered by Zhao et al. and Gilard et al., who decided to place the LITA to LAD bypass graft on the arrested heart through full sternotomy in the majority of the patients [6, 25, 26]. Lastly, some authors prefer to perform the LITA to LAD bypass graft in a totally endoscopic, port-only fashion using totally endoscopic coronary artery bypass grafting (TECAB) [13, 24]. This most challenging form of LITA to LAD bypass grafting using robotic telemanipulation techniques was initially performed on the arrested heart with the use of peripherally introduced cardiopulmonary bypass with intraaortic balloon occlusion and cardioplegic arrest [13, 24]. A major disadvantage of this approach is the use of the heart lung machine, which increases the risk of stroke, bleeding, and an inflammatory response to surgery. The latter can be solved by using beating heart TECAB (BH-TECAB), in which CPB and its considerable drawbacks are avoided [24]. Total endoscopic completion of the LITA to LAD bypass graft on the beating heart requires an additional port subxiphoidally to place a specially designed endoscopic stabilizer, which stabilizes the heart to optimize the quality of the anastomosis [24]. This so-called beating heart totally endoscopic coronary artery bypass (BH TECAB) procedure might be the least invasive approach for coronary bypass surgery without making concessions to graft patency [24, 35–38]. However, the TECAB procedure is an extremely challenging and a potentially expensive procedure with an extensive learning curve, which may raise concerns about widespread adoption and application [11].

The postoperative LITA patency seemed to be independent of the surgical technique of LITA to LAD bypass grafting, since LITA patency has shown to be approximately equal for all surgical techniques (Table 2). The postoperative LITA patency varied between 93.0% and 100.0% (mean: 98.8% ± 2.3%). The mean in-hospital MACCE rate was 1.3% ± 1.9% (range: from 0,0% to 5.6%) with relatively high MACCE rates shown by Katz et al. (3.7%), Kiaii et al. (3.4%), Zhao et al. (4.5%) and Delhaye et al. (5.6%) [13, 14, 25, 26]. Strikingly, three of these authors (Katz et al., Zhao et al., and Delhaye et al.) performed LITA to LAD placement on the arrested heart [13, 25, 26]. The percentage of patients requiring PRBC transfusion varied considerably between 0.0% and 35.4% (mean: 13.6% ± 11.7%). The surgical technique or HCR strategy (staged versus simultaneous) used did not appear to affect the percentage of patients requiring PRBC transfusion. Overall, the 30-day mortality rate was 0.4% ± 0.8% (range: from 0.0% to 2.6%). Interestingly, higher than expected 30-day mortality rates were found in studies (Gilard et al. and Zhao et al.) using on-pump CABG to perform the LITA to LAD bypass graft in the majority of patients [6, 25]. Finally, the mean overall survival rate in hybrid treated patients was 98.1% ± 4.7% (range: from 84.8% to 100.0%). 

### 3.4. PCI Techniques and Target Vessel Revascularization

Besides the technical improvements of LITA to LAD bypass grafting, innovations occurred in the field of PCI. This development was supported by the increased rate of DES implantation in later patient series compared to earlier patient series, which used percutaneous transluminal coronary angioplasty (PTCA) only or PTCA in combination with BMS implantation. Application of drug-eluting stents should lower the restenosis rate, but their potentially beneficial effect on the target vessel revascularization (TVR) is not supported by data from the included studies (Table 2). The TVR ranged between 0.0% and 29.6% (mean: 8.6% ± 7.9%). However, the (early and late) patency rate of new generation drug-eluting stents in non-LAD lesions, provided that proper DAPT is applied, may already be superior to that of saphenous vein grafts. Hard evidence is however lacking, since a head-to-head comparison of (early and late) patency rates between DES (in non-LAD lesions) and saphenous vein grafts is not available [9]. Finally, the introduction of bioresorbable scaffold (BRS) technology may improve sustainability, safety and feasibility of future HCR interventions. The application of BRS technology can make long-term DAPT redundant reducing bleeding complications without increasing the risk of stent thrombosis and may allow future reinterventions or reoperations on the same vessel if necessary due to its bioresorbable features [39].

### 3.5. HCR Procedure versus On- or Off-Pump CABG

A relatively small number of studies in our sample (Table 5) compared the HCR procedure using minimally invasive LITA to LAD bypass grafting with conventional CABG or off-pump coronary artery bypass (OPCAB) [7, 12, 27, 28]. All four of these studies selected matched controls who had undergone elective CABG or OPCAB with LITA and saphenous vein grafts through median sternotomy during the same period using propensity score matching [7, 12, 27, 28]. Kon et al. and Hu et al. found that patients in the hybrid group had a statistically significant shorter hospital length of stay, ICU length of stay, and intubation time compared with OPCAB, while de Cannière et al. reported that hospital and ICU length of stay was statistically shorter in hybrid treated patients compared with patients treated with CABG [7, 12, 28]. Halkos et al. showed that intubation time, ICU, and hospital length of stay were similar between the hybrid and OPCAB group [27]. Moreover, these studies revealed that PRBC transfusion requirements were reduced by the hybrid approach [12, 27, 28]. Lastly, the in-hospital MACCE rates were considerably lower in the hybrid groups compared with both the CABG and the OPCAB groups. 

### 3.6. Cost Effectiveness

Currently, only a few studies have explicitly explored the costs associated with hybrid coronary revascularization. De Cannière and colleagues were the first to quantify costs associated with HCR and to compare these costs with costs involved in conventional double CABG [12]. Costs were calculated using six major expenditure categories: costs of hospital admission (including intensive care unit and postsurgical cardiac ward cost as well as costs associated with delayed repeat procedures), pharmaceutical costs, surgical costs, PCI-related costs, costs of blood products, and other miscellaneous fees (including physiotherapy and consultants). The extra cost associated with PCI (including stents) in the hybrid group in comparison with the CABG group (€2.517 ± 288 versus €0 ± 0), which uses autologous grafts to treat non-LAD lesions, counterbalanced the cost savings on all other expenditure categories, which resulted in a nonsignificant cost difference at 2 years between both groups (€10.622 ± 1329 versus €9699 ± 2500; not statistically significant). It is worth mentioning that the reduced ICU and hospital length of stay due to faster recovery were largely responsible for the cost reduction in the hybrid group compared with the CABG group (€3.033 ± 499 versus €4.156 ± 1.413).

 Kon et al. showed that shorter intubation times, shorter ICU and hospital length of stay, and less PRBC transfusions resulted in a significant reduction in costs for hybrid treated patients in the postoperative period [7]. Conversely, intraoperative costs were statistically significant higher in patients undergoing HCR compared with OPCAB, largely because of longer operative times and the use of coated stents (DES) rather than autologous grafts ($14.691 ± 2.967 versus $9.819 ± 2.229; *P* < 0.001). In conclusion, the difference in intraoperative costs was almost completely outweighed by the lower postoperative costs in the hybrid group. This resulted in slightly, but not significantly, higher overall costs in the hybrid group. 

The nonhealthcare costs after HCR will presumably be lower than after CABG or OPCAB because both Kon et al. and de Cannière et al. showed that return to work was significantly faster in the hybrid group, leading to a marked reduction in absenteeism from work in hybrid treated patients [7, 12]. This difference in nonhealthcare costs should be able to compensate the opposite difference in healthcare costs, resulting in a negligible difference in total societal costs. Moreover, the emergency of simultaneous hybrid procedures in especially designed multipurpose operating rooms combining the potential of catheter-based procedures and cardiac surgery will reduce the unnecessary costs incurred by staged HCR procedures [12, 25]. Lastly, more experience with minimally invasive cardiac surgery will shorten operative times, which might help reduce total healthcare costs [7].

## 4. Discussion

### 4.1. Key Results

This review is the largest and most comprehensive report to date comparing the clinical outcomes of patients who underwent either hybrid coronary revascularization or conventional on- or off-pump CABG for multivessel coronary artery disease. Three principal findings were revealed as follows: (1) hybrid treated patients showed a significantly faster recovery with lower PRBC transfusion requirements and less in-hospital major adverse cardiac and cerebrovascular events than patients treated by on- or off-pump CABG; (2) staged procedures were associated with considerable period of times between both procedures, leaving patients incompletely revascularized and in theory at risk for cardiovascular events for a considerable length of time; and (3) the invasiveness of surgical LITA to LAD bypass grafting appeared to influence the clinical outcome, with higher MACCE and 30-day mortality rates in patients treated by more invasive surgical techniques using CPB and/or median sternotomy.

### 4.2. Limitations

As with any review, this report shares the limitations of the original studies. First, the initial reports especially included a relatively small number of patients, which may have resulted in biased results due to outliers. Furthermore, almost all studies were performed retrospectively with inherent patient selection bias, since the decision to perform the HCR procedure was taken on an individual and highly selective basis according to cardiac surgeon and interventional cardiologist discretion. Likewise, the inclusion and exclusion criteria used to select high-risk patients for the HCR procedure differed notably between the included studies, yielding a very heterogenic population. In addition, the used surgical techniques to perform the LITA to LAD bypass graft varied considerably, with learning curve issues and different levels of expertise and equipment. All these factors potentially contribute to heterogeneity, which may reduce the certainty of the evidence presented in this review. Moreover, the mean length of followup was generally short, almost never exceeding two years, which made it difficult to assess long-term clinical outcomes of hybrid treated patients. Therefore, this review relies mainly on in-hospital and short-term outcomes to assess the safety and feasibility of the HCR procedure. Another limitation was the lack of long-term systematic and routine angiographic followup of graft and stent patency in the majority of studies included in the present review, which precluded any conclusions about the graft and stent longevity of the HCR procedure. Furthermore, the comparative studies lacked randomization and nonblinded assessment of outcome, which might have led to selection bias and might have influenced outcome measures by preconceived notions about the superiority of the HCR procedure. Finally, postoperative pain, which might be higher in patients treated with conventional MIDCAB, was not included as outcome measure in the present review, because only a limited number of studies assessed this outcome measure. Notwithstanding these weaknesses and limitations, this review selected the best evidence currently available to give a broad and comprehensive overview of the preliminary results of the HCR procedure. 

### 4.3. Recommendations for Future Research

Larger, multicenter, prospective, randomized trials with long-term clinical and angiographic followup and cost analysis comparing HCR with both conventional on-pump and off-pump CABG or multivessel PCI will be necessary to further evaluate whether this hybrid approach is associated with similar promising long-term results. In the meantime, the first prospective, randomized pilot trial to compare HCR with conventional CABG in patients with multivessel coronary artery disease has been started [40]. These data are also needed to identify patient populations that would benefit most from this hybrid approach. Furthermore, more insights in the different surgical techniques for LITA to LAD bypass grafting and their clinical outcomes are necessary. Therefore, the different surgical techniques for LITA to LAD bypass grafting in the HCR procedure should be integrated in these large, multicenter HCR studies in order to determine the best way of LITA to LAD bypass grafting in HCR. Moreover, different HCR strategies (staged versus simultaneous) should be compared to decide which strategy will serve which patients best. Finally, the advantages and disadvantages of a hybrid operative suite need to be explored further. 

## 5. Conclusions

The large variability in HCR techniques makes it difficult to draw firm conclusions from the currently available evidence, but HCR appears to be a promising and cost-effective alternative for CABG in the treatment of multivessel coronary artery disease in a selected patient population. The HCR procedure was associated with short hospital stays (including ICU stay and intubation time), low MACCE and 30-day mortality rates, low PRBC transfusion requirements and TVR, high postoperative LITA patency rates, and high survival rates. These promising early outcomes warrant further research with larger sample size, multicenter RCTs to determine the definite place of HCR in the current therapeutic armamentarium against coronary artery disease. Until then, this review justifies the continued use of the hybrid approach, but careful patient selection and close cooperation between cardiac surgeons and interventional cardiologists will determine the clinical outcomes to a significant extent.

## Figures and Tables

**Table 1:** Overview of 18 series describing hybrid coronary revascularization.

Author	Date	*N*	Age (years)	Followup (months)	Strategy	Surgical procedure	PCI
Zenati et al. [17]	1999	31	69 (46–86)	10.8 ± 3.8	Staged	Open MIDCAB	PTCA/BMS
Lloyd et al. [18]	1999	18	63.2 (35–87)	6 (5–8)	Simultaneous (4) and staged (14)	Open MIDCAB	PTCA/BMS
Wittwer et al. [19]	2000	35	56.7 ± 17	11.4 ± 7.7	Staged	Open MIDCAB	PTCA/BMS
de Cannière et al. [12]	2001	20	62 ± 9	24.0	Staged	Open MIDCAB	PTCA/BMS
Riess et al. [20]	2002	57	65.7 ± 7.9	23.2 ± 8.7	Staged	Inversed L-shaped ministernotomy	PTCA/BMS
Stahl et al. [21]	2002	54	62.4 (36–86)	11.6 (1–23)	Staged	Robotic endo-ACAB	PTCA/BMS
Cisowski et al. [22]	2002	50	54.8 ± 20.1	3–32	Staged	Thoracoscopic endo-ACAB	PTCA/BMS
Davidavicius et al. [11]	2005	20	65 ± 9	19 ± 10	Staged	Robotic endo-ACAB	BMS/DES
Katz et al. [13]	2006	27	59.8 ± 8.9	3.0	Simultaneous (4) and staged (23)	Arrested-heart TECAB	BMS/DES
Us et al. [23]	2006	17	63.1 ± 20.9	21.3 ± 6.5	Staged	Reversed J-shaped inferior ministernotomy	PTCA/BMS
Gilard et al. [6]	2007	70	68.5 ± 10	33 (2–70)	Staged	On-pump (64) or off-pump (6) CABG	Stent to RCA
Kon et al. [7]	2008	15	61 ± 10	12.0	Simultaneous	Open MIDCAB	DES
Kiaii et al. [14]	2008	58	59.9 ± 11.7	20.2 (1.1–40.8)	Simultaneous	Robotic endo-ACAB	BMS/DES
Holzhey et al. [24]	2008	117	64.6 ± 12.0	21.3	Simultaneous (5) and staged (112)	Open MIDCAB (107); beating-heart TECAB (8); arrested-heart TECAB (8)	DES/BMS
Zhao et al. [25]	2009	112	63 (32–85) (median)	NR	Simultaneous	On-pump (90) or off-pump (22) CABG	DES/BMS
Delhaye et al. [26]	2010	18	62 (55–77) (median)	12.0	Staged	On-pump (13) or off-pump (5) CABG	DES
Halkos et al. [27]	2011	147	64.3 ± 12.8	38.4 (median)	Mainly staged	Thoracoscopic endo-ACAB and robotic endo-ACAB	DES
Hu et al. [28]	2011	104	61.8 ± 10.2	18 ± 7.9	Simultaneous	Reversed J-shaped inferior ministernotomy	PTCA/BMS/DES

Unless otherwise indicated, data are expressed as mean ± standard deviation. *N*: number; PCI: percutaneous coronary intervention; MIDCAB: minimally invasive direct coronary artery bypass; PTCA: percutaneous transluminal coronary angioplasty; BMS: bare metal stent; endo-ACAB: endoscopic atraumatic coronary artery bypass; DES: drug-eluting stent; TECAB: totally endoscopic coronary artery bypass; NR: not reported; CABG: coronary artery bypass grafting; RCA: right coronary artery.

**Table 2:** Outcomes of 18 series describing hybrid coronary revascularization.

Author	MACCE (%)	PRBC (%)	LITA patency (%)	Hospital LOS (days)	TVR (%)	30-day mortality (%)	Survival (%)
Zenati et al. [17]	0 (0.0)	2 (6.5)	100.0	2.7 ± 1.0	9.6	0.0	100.0
Lloyd et al. [18]	0 (0.0)	1 (5.6)	100.0	5 ± 1.5	0.0	0.0	100.0
Wittwer et al. [19]	0 (0.0)	1 (2.9)	100.0	7.5 ± 4	NR	0.0	100.0
de Cannière et al. [12]	0 (0.0)	0 (0.0)	100.0	6.7 ± 0.7	15.0	0.0	100.0
Riess et al. [20]	0 (0.0)	2 (3.5)	97.2	5.7 ± 1.8	15.8	0.0	98.2
Stahl et al. [21]	0 (0.0)	16 (29.6)	100.0	3.54 (2–12)	1.9	0.0	100.0
Cisowski et al. [22]	0 (0.0)	2 (4.0)	98.0	4.4 ± 1.7	12.7	0.0	100.0
Davidavicius et al. [11]	0 (0.0)	5 (25.0)	100.0	8.1 ± 1.6	0.0	0.0	100.0
Katz et al. [13]	1 (3.7)	NR	NR	NR	29.6	0.0	100.0
Us et al. [23]	0 (0.0)	1 (5.9)	NR	5.3 ± 1.4	17.6	0.0	100.0
Gilard et al. [6]	1 (1.4)	12 (17.1)	NR	NR	4.3	1.4	98.6
Kon et al. [7]	0 (0.0)	NR	100.0	3.7 ± 1.4	6.7	0.0	100.0
Kiaii et al. [14]	2 (3.4)	9 (15.5)	93.0	4.3 ± 1.42	5.2	0.0	100.0
Holzhey et al. [24]	3 (2.6)	NR	NR	NR	4.3	1.7	84.8 at 5 years
Zhao et al. [25]	5 (4.5)	NR	NR	6 (1–97) (median)	NR	2.6	NR
Delhaye et al. [26]	1 (5.6)	2 (11.1)	NR	10.0 (10.0–11.2) (median)	5.6	0.0	100.0
Halkos et al. [27]	3 (2.0)	52 (35.4)	NR	6.6 ± 6.7	8.8	0.7	86.8 at 5 years
Hu et al. [28]	0 (0.0)	30 (28.8)	NR	8.2 ± 2.6	1.0	0.0	100.0

Unless otherwise indicated, data are expressed as mean ± standard deviation or number (%). MACCE: major adverse cardiac and cerebrovascular events; PRBC: packed red blood cells; LITA: left internal thoracic artery; LOS: length of stay; TVR: target vessel revascularization; NR: not reported.

**Table 3:** Two-stage HCR procedure, LITA to LAD bypass grafting followed by PCI (*n* = 322).

Author	Number	Delay (mean ± SD or median)	Range
Zenati et al. [17]	29	NR	From 0–4 days
Lloyd et al. [18]	14	NR	From 1–3 days
Wittwer et al. [19]	35	7 days (median)	From 1–54 days
de Cannière et al. [12]	11	NR	From 2-3 days
Riess et al. [20]	53	4.7 ± 0.8 days (mean ± SD)	From 2–7 days
Stahl et al. [21]	35	16 days (mean)	From 18 hours to 3 months
Cisowski et al. [22]	50	6.5 ± 4.6 days (mean ± SD)	NR
Davidavicius et al. [11]	6	NR	From 2–180 days
Katz et al. [13]	12	16 days (mean)	From 2–60 days
Holzhey et al. [24]	59	NR	From 2–45 days
Delhaye et al. [26]	18	41 hours (median)	From 37–44 hours

SD: standard deviation; NR: not reported.

**Table 4:** Two-stage HCR procedure, PCI followed by LITA to LAD bypass grafting (*n* = 200).

Author	Number	Delay (mean ± SD)	Range
Zenati et al. [17]	2	NR	From 1-2 days
de Cannière et al. [12]	9	NR	From 1-2 days
Riess et al. [20]	4	22 days (mean)	From 1–63 days
Stahl et al. [21]	19	15 days (mean)	NR
Davidavicius et al. [11]	14	NR	From 2–83 days
Katz et al. [13]	12	38 days (mean)	From 2–137 days
Us et al. [23]	17	NR	Within 3 hours
Gilard et al. [6]	70	16 ± 2 hours (mean)	NR
Holzhey et al. [24]	53	NR	From 4–6 weeks

SD: standard deviation; NR: not reported.

**Table 5:** Comparison of hospital outcomes.

Outcome	de Cannière et al. [12]		Kon et al. [7]		Halkos et al. [27]		Hu et al. [28]	
	Hybrid (*n* = 20) Mean ± SD or no. (%)	CABG (*n* = 20) Mean ± SD or no. (%)	Hybrid (*n* = 15) Mean ± SD or no. (%)	OPCAB (*n* = 30) Mean ± SD or no. (%)	Hybrid (*n* = 147) Mean ± SD or no. (%)	OPCAB (*n* = 588) Mean ± SD or no. (%)	Hybrid (*n* = 104) Mean ± SD or no. (%)	OPCAB (*n* = 104) Mean ± SD or no. (%)
Hospital LOS (days)	6.7 ± 0.7*	9.0 ± 1.2*	3.7 ± 1.4**	6.4 ± 2.2**	6.6 ± 6.7	6.1 ± 4.7	8.2 ± 2.6∗	9.5 ± 4.5∗
ICU LOS (hours)	20.2 ± 1.8*	26.6 ± 11.2*	23.5 ± 10.1**	58.1 ± 37.7**	57.4 ± 145.0	52.7 ± 87.8	34.5 ± 35.6**	55.3 ± 46.4**
Intubation time (hours)	NR	NR	1.3 ± 3.4**	20.6 ± 25.7**	17.0 ± 30.8	22.7 ± 89.5	11.6 ± 6.3*	13.8 ± 6.8*
PRBC transfusion	0 (0.0)	4 (20.0)	NR	NR	52 (34.4)**	329 (56.0)**	30 (28.8)**	54 (51.9)**
In-hospital MACCE	0 (0.0)	2 (10.0)	0 (0.0)*	7 (23.3)*	3 (2.0)	12 (2.0)	0 (0.0)	0 (0.0)
Death	0 (0.0)	0 (0.0)	0 (0.0)	0 (0.0)	1 (0.7)	5 (0.9)	0 (0.0)	0 (0.0)
Stroke	0 (0.0)	0 (0.0)	0 (0.0)	1 (3.3)	1 (0.7)	4 (0.7)	0 (0.0)	0 (0.0)
MI	0 (0.0)	2 (10.0)	0 (0.0)	6 (20.0)	1 (0.7)	3 (0.5)	0 (0.0)	0 (0.0)

Conclusion	Favours hybrid		Favours hybrid		Favours hybrid		Favours hybrid	

*P value <0.05.

**P value <0.005.

CABG: coronary artery bypass grafting; OPCAB: off-pump coronary artery bypass; SD: standard deviation; LOS: length of stay; ICU: intensive care unit; NR: not reported; PRBC: packed red blood cells; MACCE: major adverse cardiac and cerebrovascular events; MI: myocardial infarction.
